# A perspective and demonstration of an approach to screening synthetic nucleic acid orders containing short nucleic acid sequences

**DOI:** 10.3389/fbioe.2026.1812800

**Published:** 2026-05-21

**Authors:** Bryan T. Gemler, Kevin Flyangolts, James Diggans, Bruce J. Wittmann, Patrick A. Fullerton, Meghan J. Seltzer, Craig Bartling

**Affiliations:** 1 Battelle Memorial Institute, Columbus, OH, United States; 2 Aclid, New York, NY, United States; 3 Twist Bioscience Corporation, South San Francisco, CA, United States; 4 Microsoft Corporation, Carpinteria, CA, United States; 5 MITRE, Washington, DC, United States

**Keywords:** biosecurity, DNA, olio pool, risk, synthetic nucleic acids

## Abstract

Screening of nucleic acid synthesis orders is expanding to include short, single-stranded DNA orders in efforts to ensure potentially harmful genes and genomes cannot be synthesized by illegitimate or irresponsible customers. Short sequence fragments are not as information rich as gene sequences, typically require assembly to be functional, and are much cheaper to produce than genes. Thus, here we provide the perspective that the screening of sequence fragments requires a different approach than sequence-by-sequence screening by considering the context of other fragments in the order or across orders. Additional and alternative metrics should be included while screening fragments, such as fraction of the gene/genome covered and evidence of potential use for assembly to enable efficient and accurate biosecurity screening. We further demonstrate two existing biosecurity tools, UltraQUICK and Aclid that provide outputs for better understanding the threat of orders containing fragments. This perspective provides a foundation for how short DNA fragments, including oligo pools, should be screened and leaves the reader with considerations for overall risk assessments of such orders based on the functional and taxonomic characteristics of the order.

## Introduction

In 2023, the US Dept of Health and Human Services (HHS) updated their nucleic acid screening guidelines and expanded scope (U.S. Department of Health and Human Services, 2023), heightening interest in screening across the US government, industry, and related authoritative entities. In 2024, the Office of Science and Technology Policy published and subsequently revised a Framework for Nucleic Acid Screening (Office of Science and Technology Policy, 2024), the IGSC updated its harmonized screening protocol (The International Gene Synthesis Consortium, 2024), and the International Organization for Standardization published ISO standard 20688-2:2024, which includes stipulations for sequence screening (The International Organization for Standardization, 2024). In 2025, the White House published an Executive Order calling on the revision or replacement of the OSTP Framework to strengthen oversight, increase accountability, clearly define the scope of covered research, and incorporate enforcement mechanisms to ensure compliance (The White House, 2025), but no updates have been published as of the time of this writing. Also in 2025, a bill was introduced into Congress to authorize continued work on standards for sequence screening (US House of Representatives, 2025), and later in 2025, the National Institute of Standards and Technology (NIST) published a comparison of various screening tools against a standardized sequence test set (Laird et al., 2025). Most recently, another bill has been introduced into Congress regarding sequence screening (Biosecurity Modernization and Innovation Act of 2026, 2026).

While each of these publications provides different context, granularity, and authority, some common trends among them include striving toward screening for sequences that are: 1) sequences of concern (i.e., that contribute directly to pathogenicity or toxicity), 2) either single- or double- stranded, 3) short (e.g., 50 nucleotides or less), and 4) potentially split across orders and/or vendors. While seemingly trivial, these trends are a departure from 2010 HHS guidelines (U.S. Department of Health and Human Services, 2010) and US export control guidelines (Bureau of Industry and Security, 2026), which focus on screening double stranded and longer DNA sequences only (i.e., genes or sequences 200 bp or more). Importantly, while both short ssDNA and long dsDNA are nucleic acid products; long, dsDNA (and RNA) can be directly used to express proteins (the biological macromolecule that typically carries out the function), whereas short, ssDNA typically needs to be assembled and amplified into dsDNA prior to use in protein expression.

Based on previous guidelines, current screening tools were built for sequence-by-sequence screening of dsDNA (i.e., genes). These screening tools strive to minimize both false negatives and false positives to ensure high biosecurity standards and low overhead screening burden, respectively. While screening approaches vary, all publicly available tools provide some sort of flag for sequences that require manual review and potential customer follow-up. Notably, dsDNA gene orders have a much higher price point [(e.g., ∼$0.23 per base pair (IDT, 2026) compared to large ssDNA oligo pools (e.g., >600,000 oligos, which can sell for <$0.0006 per base) (Twist Bioscience, 2026)], emphasizing the need for low overhead (minimal false positives) when screening ssDNA pools. While one gene requires only screening one sequence, an oligo pool may require screening hundreds of thousands or even millions of sequences. Thus, while biosecurity flag rates of 1%–5%, for example, may be economically viable for dsDNA orders, those same rates are likely not reasonable for ssDNA orders. Further yet, flag rates will typically increase as sequence length decreases due to a higher potential of random hits [e.g., “Best Matches” as defined in (U.S. Department of Health and Human Services, 2023)] during database searches. For example, we have shown that moving from 200 nt to 50 nt causes an increased flag rate of ∼2% (Gemler et al., 2024), which can equate to tens of thousands of sequences in a single large ssDNA order.

Given that ssDNA oligos require assembly to be functional, as well as the commercial reality that they are much lower-margin products that gene-length nucleic acids (meaning that far less resources are available to screen each order for potential misuse), the following critical question arises when screening these products - should short, ssDNA orders be “flagged” in the same sequence-by-sequence manner as dsDNA (i.e., genes)? In other words, do ssDNA orders have the same threat profile as genes? Here we argue that the threat associated with a sequence fragment (i.e., sub-gene or sub-functional sequence) is different than genes and that the threat should be considered in the context of other sequences in the order and possibly across orders. We provide the perspective that the screening of sequence fragments only requires lightweight bioinformatics tools that can handle large volumes of sequences, but these tools should provide evidence (or lack thereof) of the potential use for assembly into genes and organisms and completeness. We demonstrate two example tools, UltraQUICK and Aclid, which provide outputs for understanding the threat of fragments contained within an order and across orders.

## What information is important to consider when screening fragments/Oligo pools?

### Taxonomic identification

Some of the same considerations for screening genes are relevant to fragments as well. Determining which organism the sequence is a Best Match to or unique to can be critical in ultimately building toward an overall risk profile. Note that we do not intend to debate which organisms to flag (beyond the scope of this perspective), rather, we simply note that determining taxonomic origin and specificity of the sequence is important.

### Function

We and others have published on the types of threatening functions to be considered (Gemler et al., 2022; Godbold et al., 2022), and the specific functions are beyond the scope of this perspective as well. Rather, here we argue that whether or not the protein encoded by the sequence would be functional at all is critical to a risk profile. In contrast to full length genes that encode functional proteins, smaller sequences may be fragments of genes that may or may not encode (or nearly encode) a functional protein. A very conservative estimate would be to assume that if <50% of the gene is present, then the expressed protein would not be functional. We base this estimate on data around maintenance of function for known pseudogenes and publicly available mutagenesis data sets. We note that viral polyproteins and subunits of toxins may still retain activity with <50% of an open reading frame present, so here we only focused on non-viral and non-toxin genes for this conservative estimate. However, toxin and viral genes are critical to screening efforts as elaborated later in this perspective. Specifically, Zhang et al. (2003) cataloged human pseudogenes, noting that even though human pseudogenes are on average 94% complete in coding regions with 75% similarity at the amino acid level, these sequences are non-functional. Feng et al. (2022) further showed that expression of bacterial pseudogenes are on average, 10% of their intact homologs. In regard to mutagenesis data, we searched UniProt [(ft_mutagen:*) AND (taxonomy_id:2) OR (taxonomy_id:4751)] for accessions from bacteria or fungi in which experimental evidence of mutating at least two consecutive amino acids is documented (i.e., not just point mutants). The search resulted in 1,158 proteins and 2,268 experiments, and a review of the assertions and sequence lengths revealed only a single UniProt accession (a structural protein in cyanobacteria that enabled gas vesicle stability) in which <50% of the length of the protein remained but near wild-type activity was reported. Thus, given the lack of public data supporting maintenance of activity in such truncated sequences, the 50% threshold is a reasonable starting point to assume lack of activity for non-viral and non-toxin genes.

### Coverage and assembly

Gene orders are typically synthesized from the assembly of short ssDNA fragments. Full genomes can similarly be constructed from a collection of shorter sequence components. Thus, another important metric when considering the risk profile of an oligo pool order is total coverage of ordered sequences at the gene and genome level. For example, is >50% of the gene covered as described above? And if so, how many genes in the genome are so covered in the order? And since the goal is to identify sequences of concern, we can further evaluate the percent of sequences of concern that are covered at some threshold in the order?

Beyond coverage, screening tools should strive to determine possibility of assembly. Consider the following 2 ssDNA oligo pool order scenarios. In the first scenario, an order contains 4 sequences of 50 nucleotides each covering non-overlapping segments of the same 500 nucleotide-length sequence of concern (40% coverage). In the second scenario, an order contains 4 different sequences of 50 nucleotides each that cover segments of the same 500 nucleotide-length sequence of concern, but they overlap with each other to cover nearly 40% of the same sequence of concern. We argue that the order in scenario 2, as opposed to scenario 1, would be considerably better suited for assembly of the sequence of concern, which should be taken into consideration when evaluating risk.

## Example screening tool outputs for oligo pools

Two simple and efficient commercially available screening tools (Aclid and UltraQUICK) enable screening of fragments at the order level (and/or across orders) and provide metrics useful for oligo pool screening as discussed in the previous section. The Aclid tool was previously part of NIST’s Inter-tool comparison and is further described in Laird et al. (2025). Briefly, Aclid employs a sequence-based approach using reference sequences and annotations derived from public and proprietary experimental, simulated, and predicted datasets. The cloud-based screening system includes curated databases of sequences and functional annotations, hardware-accelerated sequence similarity and alignment tools, and algorithms to interpret compliance requirements from sequence data.

The screening system flags a Best Match to a regulated agent in a 50-nucleotide or 16-amino acid window. Shorter Best Matches to a regulated agent are aggregated and distinct nucleotides or amino acids exceeding a 50-nucleotide or 16-amino acid window, respectively, are also flagged. In addition to flag results, the screening system includes:

- Alignment metrics and functional data for each match in a sequence
- Aggregated gene and genome coverage across sequences for each species and regulated agent
- Statistical summary of the sequence flags across regulatory agents and frameworks

UltraQUICK is a flexible, light weight companion tool to UltraSEQ; the latter was part of and described in the Laird et al. (2025) study and other studies (Gemler et al., 2024; Gemler et al., 2023; Wheeler et al., 2024; Wittmann et al., 2025). UltraQUICK focuses on faster, order-level metrics as opposed to sequence-level metrics. UltraQUICK runs on a similar AWS-GovCloud infrastructure as UltraSEQ, but is built to have a much quicker response time. UltraQUICK leverages the same Sequence of Concern (SoC) database (Gemler et al., 2022) as UltraSEQ, proteomes from model organisms, and a curated Uniref100 database that includes UniRef100 protein sequences in which the seed TaxID is from a controlled organism [e.g. (Bureau of Industry and Security, 2026), and related lists] and their near neighbors, plus “translated” internal ribosome entry sequences. UltraQUICK uses an efficient logic scheme in which alignments against these databases [using the lambda2 aligner (Hauswedell et al., 2014)] are processed as follows:

- Any alignment <30 nt is filtered out
- Expect (E)-value thresholds of 100 and 1 for the curated Uniref100 and SoC databases, respectively, based on empirical testing with the NIST standard dataset as described in Laird et al. (Laird et al., 2025)
- Percent identity thresholds of 80% and 45% for the curated Uniref100 and SoC databases, respectively
- Hits whose Best Match is to a controlled TaxID are compiled
- For each controlled protein, a tiling detection algorithm is implemented to search for overlapping queries with at least seven nucleotides of overlap

Hits are compiled into coverage assessments organized at the taxonomic level for each controlled taxa (and toxin) identified in the order, which are provided in a json-style report. Results at the TaxID level are summarized for both hits to the curated UniRef100 database (UniRef) and SoC database. These metrics include:

- Number of query hits = the number of query sequence hits to accessions from the specific controlled organism TaxID (as described above for the UltraQUICK tool)
  - Fraction of query hits = the number of query hits divided by the total number of input queries.
- Number of UniRef or SoC hits = the number queries that hit to UniRef or SoC accessions in the UltraQUICK database for the controlled organism TaxID
  - Fraction of UniRef or SoC hits = number above divided the total number of UniRef or SoC entries in the database for the controlled organism TaxID
- Fraction of UniRef or SoC hits with at least 50% protein coverage = same as the Fraction defined in the immediate sub-bullet above, except only hits in which at least 50% of the subject is covered in the alignment are included
- Average and variance of protein coverage across UniRef and SoC hits = the mean subject coverage for UniRef/SoC alignments and variance for the mean
- Fraction of UniRef and SoC hits with at least one detected tiling event = number of UniRef/SoC hits in which at least seven nucleotides overlap divided by total number of UniRef or SoC entries in the database for the controlled organism TaxID

Since UltraQUICK was not part of the Laird et al. (2025) study, we validated UltraQUICK using the same dataset (available here: https://data.nist.gov/od/id/mds2-3787). The results show that UltraQUICK has the following performance metrics: FP Rate: 0.054, FN Rate: 0.020, Accuracy: 0.963, and Sensitivity: 0.980. These results are in line with other tools (Laird et al., 2025), thus demonstrating UltraQUICK as valid tool for sequence screening in general. Notably, the only NIST-defined FNs observed were non-coding genes from *Coxiella burnetti*, which were classified as non-threats according to the framework used in this paper.

## Demonstration of two screening tools for oligo pool analysis

**FIGURE 1:**
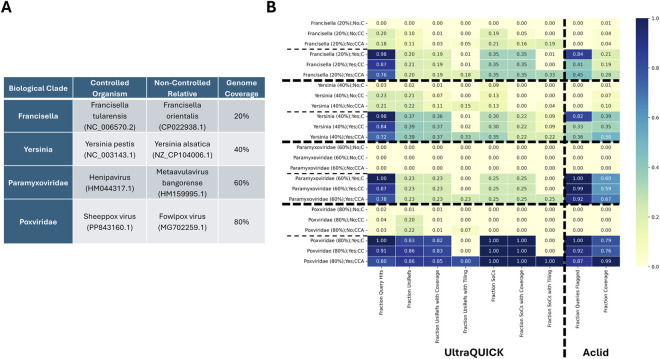
**(A)** Dataset composition. **(B)** UltraQUICK and Aclid performance. Panel **(A)** outlines the specific organisms and accessions for the controlled and non-controlled dataset, and what percentage of the genome (last column) was used as input for testing. In Panel **(B)**, each row represents the results of the dataset run through UltraQUICK and Aclid. The label for each row (i.e., the first column) consists of the organism group, genome coverage of the initial dataset, whether or not the dataset is from the controlled agent (“Yes”) or non-controlled agent (“No”), and the dataset type, where “C” = the original “chunked” dataset of 1,000 nts, “CC” = the 51-mer chunks derived from the 1,000 nt chunks, and “CCA” = 1,000-mer chunks run through the Oligo assembly tool. Column headings are provided in the “Example Screening Tool Outputs for Oligo Pools” section.

To demonstrate the efficacy of these two bioinformatic tools for analyzing oligo pools, we developed a “controlled/non-controlled agent” dataset. This dataset includes eight genomes (4 controlled TaxIDs and 4 related non-controlled TaxIDs) with varying genome coverages. For each genome, three sub-datasets were created: i) 1,000 nucleotide non-overlapping segments, ii) 51 nucleotide non-overlapping segments, and iii) an oligo-designed dataset generated using the “oligoOptimizer” tool from (Liang et al., 2022) where the tool was run on the 1,000 nucleotide segments to design feasible oligos for assembly. The dataset construction is shown in Figure 1A.

We found during testing that generation of the oligo assembly fragments from the 1,000 nt chunks using the oligoOptimizer tool resulted in a distribution of lengths around the input (51), with ∼95% above 30. The 5% below 30 nt are the primer sequences, which are shorter by design and will thus not be flagged by any tool that has a threshold <30 nt.

The results of UltraQUICK and Aclid with the controlled/non-controlled agent dataset are shown in Figure 1B. The results show the query hits to the controlled agent from which the sequences were derived from (e.g., *Francisella tularensis* for both the controlled and non-controlled *Franscisella* datasets; *Yersinia pestis* for both the controlled and non-controlled *Yersinia* datasets, see Figure 1A).

The results demonstrate that across both oligo screening tools, the fraction coverage of controlled agent (represented in UltraQUICK by UniRef clusters or SoCs hit (with or without coverage) and in Aclid by a fraction genome coverage) are generally much higher for the controlled agent compared to the non-controlled agent datasets for all three dataset types (1,000-mer, 51-mer, and oligo assembled). The fraction coverage metrics for the controlled organisms are roughly correlated with the genome coverage as defined in Figure 1A. For example, the genome coverage for *Yersinia* (40%, Figure 1A) approximately matches UltraQUICK’s Fraction Uniref and Fraction SoC coverage and Aclid’s Fraction Coverage (Figure 1B). For metrics from non-controlled datasets, some values are >0 due to differences in tool designs such as protein vs. nucleotide reference databases, sequence uniqueness calculations, and overall tool logic. Additional testing and truth dataset generation may be required to completely tune tools.

The results also demonstrate that tiling metrics can be used to differentiate datasets that have indication of oligo assembly compared to those that do not (i.e., compare the “Fraction UniRefs with Tiling” or “Fraction SoCs with Tiling” values for the “CCA” datasets compared to the “C” and “CC datasets”). While a specific threshold for these values is dependent on the risk tolerance of the user of the tool, area under the curve-type of analyses could be performed to select metric thresholds for flagging an oligo pool order as a threat versus nonthreat, allowing for the effective quantification of false positive versus false negative tradeoffs.

## Discussion

**FIGURE 2:**
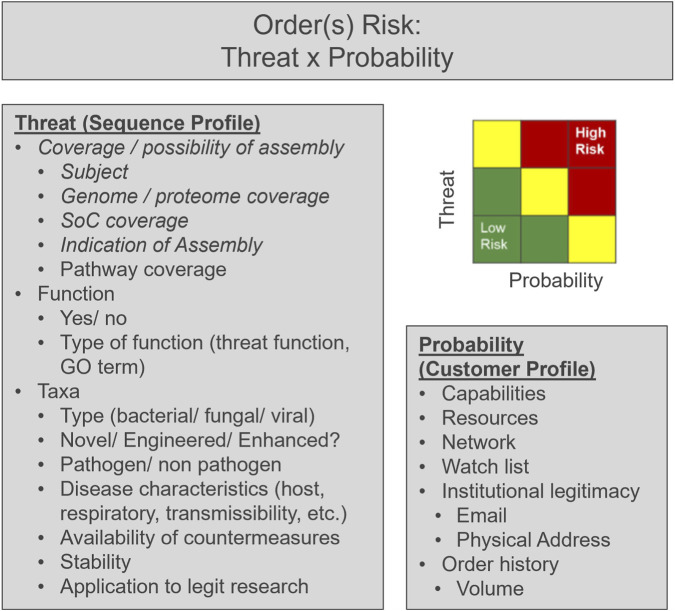
Considerations for risk assessment of nucleic acid orders. When considering the overall risk of a collection of nucleic acid sequences (either within an order or possibly across orders), risk is dependent on both the sequence profile and the customer profile. Example parameters to consider are shown in the figure, with parameters that are the focus of this perspective in *italics*.

Here we present the perspective that screening sequence fragments (oligo pools) is different than gene-by-gene screening and demonstrate two screening tools that provide outputs for understanding risks at the order level by analyzing all the sequences in the order as a whole. To that end, a similar approach could be used to analyze across order streams in cases where split order assembly is suspected. Regardless, this perspective can be leveraged by nucleic acid providers and consumers for biosecurity purposes, but we recognize that understanding the contents of a nucleic acid order provides only a foundational piece to understanding its overall risk. While a full-blown risk assessment is well beyond the scope of this perspective, we leave the reader with potential considerations as inputs for the risk assessment of nucleic acid orders that could be implemented at the single vendor or multi-vendor level. For example, the overall risk profile of an order (or orders) should consider the threat potentially being assembled as well as the risk profile of customer placing the order(s) (Figure 2).

We argue that for a sequence to be a “threat,” the sequence must be functional (i.e., encode a complete functional protein). For most sequences, except toxins and genes that endow or enhance pathogenicity when transferred to another organism, the ultimate “function” is the organism itself, which is built from its genetic components. Under this framework, a risk assessment should take into consideration the function of specific genes and characteristics of an organism to understand overall risk. For example, what specific threat functions are contained in the order (e.g., toxins, immune evasion proteins, etc.)? And if a genome is being assembled, what are the characteristics of the organism (disease causing, transmissible, etc.)? We show here that using the context of the order or orders, coverage and assembly metrics can aid biosecurity officers in understanding the likely intent of the order. For example, if an oligo pool contains 1,000 flagged sequences matching SARS-CoV-2, intent could either be (for example) a benign mutagenesis study on a specific gene or a malicious assembly of an entire genome. In the former, there would be minimal coverage to the entire SARS-CoV-2 genome with many flagged sequences, while in the latter, genome coverage should be near complete. Thus, the gene and genome-level metrics demonstrated by the tools provide a foundation for risk-based biosecurity decisions.

The threat of an order should be paired with the above sequence-assessment with information about the customer(s). For considerations of legitimacy of customers, the reader is referred elsewhere (Engineering Biological Research Consortium, 2025). Some considerations may include, for example, a customer’s resources and capabilities.

While no absolute rubric exists for how to assess risk for nucleic acid orders, this perspective details critical outputs of oligo pool screens that are useful in the context of an overall risk assessment. Notably, the test sequences used in this study were from published genomes; screening systems should be tested with sequences that are more likely to be contained within order streams (mutated sequences, designed sequences, etc.). In summary, as the community builds toward methodologies for overall risk assessments for nucleic acid orders, we strongly suggest future discussions of nucleic acid risk assessment profiles should consider threat sequence coverage and the potential for assembly from nucleic acid order streams to ensure objective, rational, and efficient biosecurity risk assessments.

## Data Availability

The original contributions presented in the study are included in the article/supplementary material, further inquiries can be directed to the corresponding authors.
